# Tissue-Specific Differences in the Spatial Interposition of X-Chromosome and 3R Chromosome Regions in the Malaria Mosquito *Anopheles messeae* Fall.

**DOI:** 10.1371/journal.pone.0115281

**Published:** 2015-02-11

**Authors:** Gleb Artemov, Semen Bondarenko, Gleb Sapunov, Vladimir Stegniy

**Affiliations:** 1 Tomsk State University, Tomsk, Russia; 2 Institute of Theoretical and Experimental Biophysics of RAS, Pushchino, Moscow region, Russia; 3 Institute for Biological Instrumentation of RAS, Pushchino, Moscow region, Russia; University of Maryland School of Medicine, UNITED STATES

## Abstract

Spatial organization of a chromosome in a nucleus is very important in biology but many aspects of it are still generally unresolved. We focused on tissue-specific features of chromosome architecture in closely related malaria mosquitoes, which have essential inter-specific differences in polytene chromosome attachments in nurse cells. We showed that the region responsible for X-chromosome attachment interacts with nuclear lamina stronger in nurse cells, then in salivary glands cells in *Anopheles messeae* Fall. The inter-tissue differences were demonstrated more convincingly in an experiment of two distinct chromosomes interposition in the nucleus space of cells from four tissues. Microdissected DNA-probes from nurse cells X-chromosome (2BC) and 3R chromosomes (32D) attachment regions were hybridized with intact nuclei of nurse cells, salivary gland cells, follicle epithelium cells and imaginal disсs cells in 3D-FISH experiments. We showed that only salivary gland cells and follicle epithelium cells have no statistical differences in the interposition of 2BC and 32D. Generally, the X-chromosome and 3R chromosome are located closer to each other in cells of the somatic system in comparison with nurse cells on average. The imaginal disсs cell nuclei have an intermediate arrangement of chromosome interposition, similar to other somatic cells and nurse cells. In spite of species-specific chromosome attachments there are no differences in interposition of nurse cells chromosomes in *An. messeae* and *An. atroparvus* Thiel. Nurse cells have an unusual chromosome arrangement without a chromocenter, which could be due to the special mission of generative system cells in ontogenesis and evolution.

## Introduction

Numerous studies confirm that each chromosome occupies a definite space, called chromosome territory [1–4]. In the last decade, the spatial organization of chromosomes is supported as an important factor in the regulation of genes and stability of the genetic apparatus [5,6]. The nonrandom position of chromosomes in the nucleus is provided by attachment of chromosomes to the frame structure, which is performed by the nuclear lamina [7,8]. It has been shown that there are domains in the genome associated with a major component of nuclear lamina—lamin B protein. Some of them have a conserved chromosome location in several tissues, but others are tissue-specific [9]. It can be explained by participation of chromosomes’ attachment regions in the process of cell differentiation. Foundation of contacts in particular chromosomal regions may cause the activation or repression of certain patterns of genes specific for each cell line. We hypothesized that the relative position of these chromosome contacts with the nuclear envelope may also be involved in the process of cell differentiation. To test this hypothesis we elected to assess the relative positions of two chromosomes in the nuclear space of cells from different tissues.

The polytene chromosomes in ovarian nurse cells of malaria mosquitoes form strong contacts with the nuclear envelope, which has been investigated in semisquashed nuclei [10–12]. It is important that the system of these contacts in ovarian nurse cells is species-specific. In malaria mosquitoes *Anopheles messeae* Fall. chromosome 3 in the pericentric region and the X-chromosome in the middle of the left arm have remarkable attachments to the nuclear envelope. In contrast, there are no such strong attachments in chromosome 2. The sibling species, *An*. *atroparvus* Thiel., reveals a similar system of chromosome architecture in ovarian nurse cells, but, in contrast, the X-chromosome is anchored on the nuclear envelope via the pericentric region. These chromosome attachments may be visualized with the use of light microscopy.

The differences in chromosome architecture in nurse cells has been described for several *Anopheles* species from the palearctic branch of the “maculipennis” complex: *An*. *messeae*, *An*. *beklemishevi* Stegn. et Kab. *An*. *atroparvus*, *An*. *maculipennis* Mg., *An*. *melanoon* Hackett., *An*. *sacharovi* Favre, *An*. *martinius* Shing. [10, 13], *An*. *labranchiae* Fall. [14], and *An*. *artemievi* Gordeev et al. [15]. The chromosome spatial organization specificity appears in three features: 1) availability or absence of strong chromosome attachments; 2) localization of these attachments on the chromosome; 3) morphological structure of chromosome attachment regions.

Chromosome attachment regions have a heterochromatic nature, that is similar to beta-heterochromatin in *Drosophila* salivary glands chromosomes [16, 11, 17]. Moreover, a high presence of repetitive DNA in the chromosome attachment region was shown by *in situ* hybridization experiments [16, 18]. The X-chromosome attachment region (2BC) was microdissected with subsequent DNA sequencing [18]. We found a high variety of transposable elements in this region, especially retrotransposons. Comparative analysis of DNA sequences from 2BC showed that many of them localize in the chromosome attachment region of the 3R chromosome *An*. *messeae* and X-chromosome *An*. *Atroparvus*, but not in the pericentric alpha-heterochromatin of *An*. *messeae* chromosome 2, which has no strong attachments to the nuclear envelope (unpublished data). Probably, the regions that provide such strong chromosome attachments have similar DNA sequences.

Immunostaining of malaria mosquitoes polytene chromosomes with anti-lamin B antibody was used as an addition marker in order to explore chromosome attachments to the nuclear envelope [17]. This approach helped us to find new interspecific differences in the X-chromosome attachment system [19]. Revealed contacts of chromosomes with the nuclear envelope are not exclusive and there are many more regions that could be discovered by more sensitive methods such ChIP [20] or DamID [21]. Nevertheless, we focused on the major and, probably, the most substantial regions for chromosome positioning. In this study, we assessed the interposition of the X-chromosome and 3R chromosome attachment regions in the nuclear envelope in different tissues of the malaria mosquito *An*. *messeae* and in nurse cells of the sibling species *An*. *messeae* and *An*. *atroparvus*. In a comparative analysis, we expected to identify differences that would indicate an importance of chromosome`s interposition for function, development of cells and in species evolution.

## Materials and Methods

### Mosquitoes collection and ovaries fixation

The imago of *An*. *messeae* malaria mosquitoes was collected in the natural population of Kolarovo village (20 km from Tomsk, Tomsk region, Russia) in a cattle shed. *An*. *atroparvus* females were obtained from a laboratory colony hosted at the Tomsk State University, Russia. Ovaries in the III development stage (when ovaries occupy one half of the female abdomen) were dissected in PBS drop and transposed in individual tubes with fresh and cooled down Cornoy`s fixative (3 ethanol: 1 glacial acetic acid by volume) at -20°C. Larvae in the IV development stage were collected from ponds surrounding Kolarovo village and fixed in Cornoy`s solution as a whole. Ovaries and larvae were preserved in fixative solution from 24 hours up to one month at -20°C.

Mosquitoes collection at Kolarovo village is not required any permits or approvals. Kolarovo village (56°20′06″ n. lat., 84°56′19″ e. long.) is situated in Tomsk region on non-protected land. *Anopheles messeae* is non-protected species with huge areal from East Siberia to West Europe. This species is usual for Siberia region and predominate within anopheline mosquitoes on this territory.

### Chromosome preparation

For one preparation of ovarian nurse cell chromosomes, a single ovary from one pair was taken. Two salivary glands were dissected for one chromosome preparation. The ovaries or salivary glands were held for 5 minutes, maturated and squashed in a drop of 50% propionic acid. The quality of the preparation was inspected with an AxioImager A1 microscope (Carl Zeiss, OPTEC Company. Siberian Office, Novosibirsk, RF). Then high quality preparations were frozen in liquid nitrogen. Preparations were dehydrated in a series of ethanol (50%, 70%, 95%) and air dried.

### DNA-probe labeling and fluorescent in situ hybridization

DNA-probes from the 2BC and 32D regions were obtained by the microdissection procedure detailed in previous works [22, 18]. DNA probe labeling was performed in a PCR reaction mix: Taq DNA-polymerase buffer (10 mM Tris HC1, 50 mM KC1, pH 8.3, SibEnzyme Ltd., Novosibirsk, Russia), 2.5 μM MgCl_2_, 200 μM dATP, 200 μM dCTP, 200 μM dGTP, 200 μM dTTP, 100 μM Tetrametilrhodamin-5-dUTP (BIOSAN, Novosibirsk, Russia) or 100 μM Biotin-11-dUTP (Fermentas, Vilnius, Lithuania), 2 μM MW6-primer (5'-CCGACTCGAGNNNNNNATGTGG-3', SibEnzyme Ltd., Novosibirsk, Russia), 1.5 units Taq DNA polymerase (SibEnzyme Ltd., Novosibirsk, Russia). Tetrametilrhodamin-5-dUTP was used for 2BC DNA-probe making, while biotin-11-dUTP was used for the 32D DNA-probe in order to simultaneous DNA-probe detection. FISH was performed using standard protocol for polytene chromosomes of anopheline mosquitoes [23]. For general chromosomes staining DAPI (Vector Laboratories Inc., USA) was used.

### Chromosome immunostaining

Preparation of polytene chromosomes from nurse cells and salivary glands were immunostained with antibody to *Drosophila melanogaster* lamin B—lamin Dm0 (ADL84.12, DSHB, USA) consistent with the tested protocol [17].

### 3D fluorescent *in situ* hybridization

DNA-probe labeling for 3D-FISH was performed as described above. For the 3D-FISH procedure, adult mosquito ovaries (for nurse cells and follicle cells analyzing), larvae salivary glands and appendages imaginal discs were dissected and placed in PBS solution (137 mM NaCl, 2.7 mM KCl, 10 mM Na_2_HPO_4_, 1,8 mM KH_2_PO_4_). Further 3D-FISH procedures were conducted according to the appropriate protocols [24, 25].

### Microscopic analysis

Chromosome preparations after FISH and immunostaining experiments were observed with the AxioImager Z1 equipped with an Apotome device for contrast enhancement and with AxioVision rel. 4.7.1 software (Carl Zeiss, OPTEC Company. Siberian Office, Novosibirsk, Russia) for image fixing and further processing. The big nuclei, such as from nurse cells, salivary glands cells and follicle cells (with an average diameter from 6 to 24 mcm) (Tables A-D in S1 File) were analyzed by AxioImager Z1 with Apotome and AxioVision rel. 4.7.1 software. Small imaginal disc nuclei (the average diameter is about 3 mcm) (Table E in S1 File) were observed with a Laser Scanning Microscope 780 and software ZEN (Carl Zeiss, Center for Collective Use of The Institute of Cytology and Genetics SB RAS, Novosibirsk, Russia).

### 3D-FISH image processing and quantitative evaluation methods

**Fig 1:**
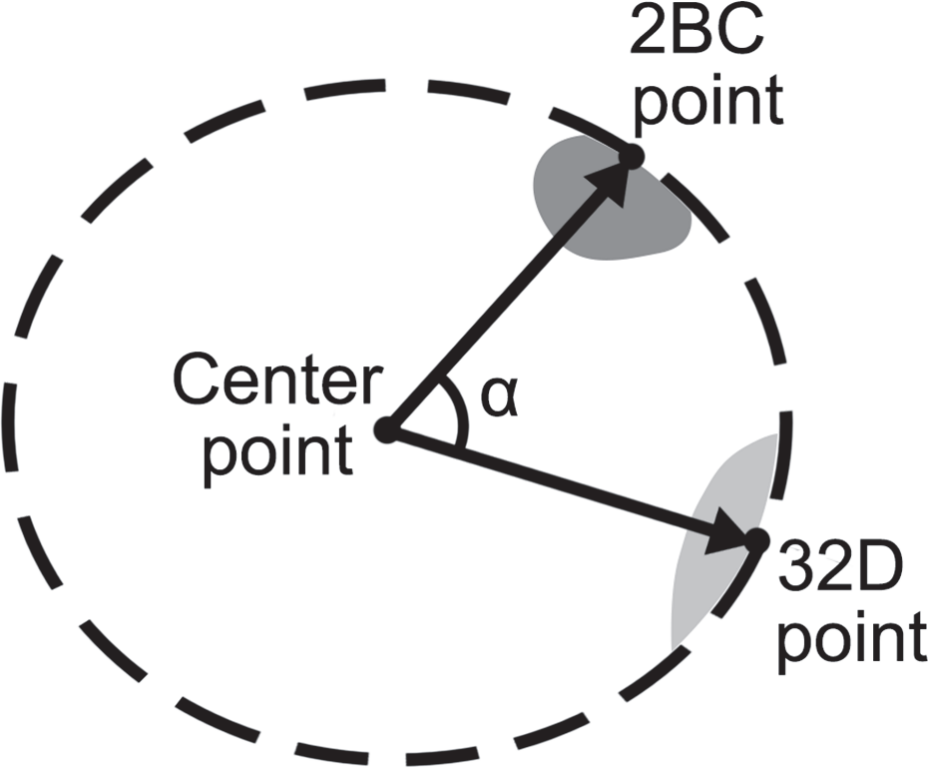
Arrangement of 32D, 2BC points and a center point in the nucleus required for analysis of distance between X-chromosome and 3R chromosome regions by α angle calculation.

In order to measure the distance between two distinct chromosome regions (2BC and 32D) we calculated the angle between two imaginary vectors from the nucleus center to a point on peripheral surface of a 2BC or 32D fluorescent signal. All 3D-FISH images were analysed using AxioVision rel. 4.7.1 software and images, which were obtained with ZEN software, were exported in zvi-format, appropriate for AxioVision rel. 4.7.1 software. The program mode “Cut view” enables the reading of the coordinates of all points of interest. Thus for every nucleus we read the X, Y and Z coordinates of the nucleus center point, 2BC point and 32D point (Fig. 1). The coordinates of each point by the Z axis underwent correction required for equalization of differences in the resolution of the image in the X0Y plane and X0Z (Y0Z) plane. For this reason the values of Z are multiplied by 6.1875 for non-confocal images or by 2 for confocal images. The coordinates for vectors were calculated as a difference of the coordinates of the 2BC or 32D points and the nucleus center point. The angle between vectors (α) was calculated by the formula:

$$\alpha =arccos\frac{X_{2BC}\times X_{32D}+Y_{2BC}\times Y_{32D}+Z_{2BC}\times Z_{32D}}{\left(X_{2BC}^{2}+Y_{2BC}^{2}+Z_{2BC}^{2}\right)\times \left(X_{32D}^{2}+Y_{32D}^{2}+Z_{32D}^{2}\right)}$$

where X_2BC_, Y_2BC_, Z_2BC_ are coordinates of imaginary vector, whose line from the nucleus center points to the 2BC signal; X_32D_, Y_32D_, Z_32D_ are coordinates of imaginary vector, whose line from the nucleus center points to the 32D signal (Tables A-E in S1 File). The **α** is an angle in degrees used as a criterion of distance between the peripheral part of signals of the 2BC and 32D DNA-probes that we obtained in the FISH experiment. For nurse cells, where 3R chromosome homologues are separated from each other we often read the coordinates of the two 32D points and calculate the angle for each homologue. For subsequent analysis we used the mean value of these angles. In general the **α** angle value connected with a distance between points of interest. Thus, the greater the angle value, the greater the distance between points. For simplification of result description we divided the row angle values on three groups and call it as *juxtaposed* (α from 0 to 59.99 degrees), *average* (α from 60 to 119.99 degrees) and *distant* (α from 120 to 180 degrees).

In addition we calculated the average diameter of nuclei for each cell type, as an average distance between the center point and the 2BC 32D (two for nurse cells and one for others) points (Tables A-E in S1 File).

### Statistical tests

Statistical tests were performed using Statistica v10 software (StatSoft, Inc., USA) and Microsoft Office Exel (Microsoft Corp., USA). For statistical comparison of angle values from one type of cells with others a t-test for independent samples and ANOVA multiple analysis were used.

## Results

### Tissue-specific organization of nurse cells chromosome attachment regions in salivary glands cells

**Fig 2:**
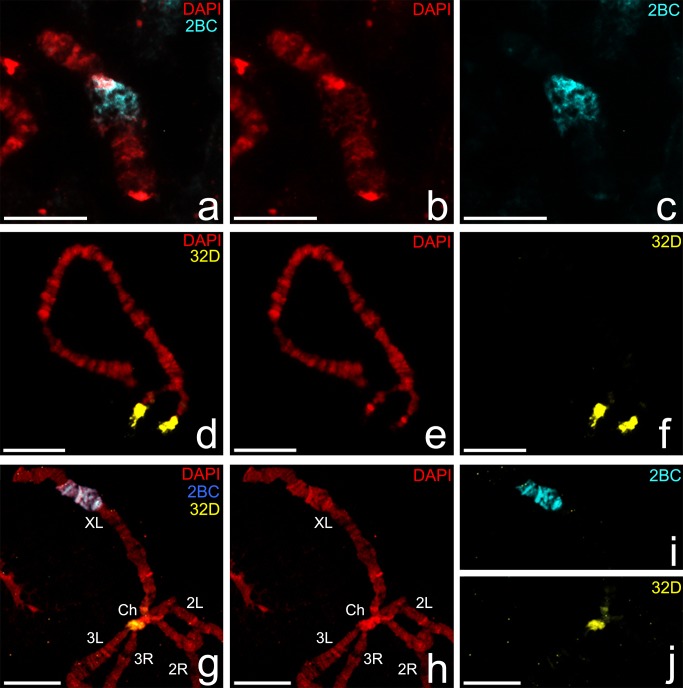
Differences in linear organization of polytene chromosome from nurse cells and salivary glands cells of *Anopheles messeae*. Localization of the DNA-probe of the X-chromosome attachment region (2BC) on the X-chromosome from nurse cells (a-c); localization of DNA-probe of the 3R chromosome attachment region (32D) on 3R chromosome (d-f); localization of 2BC and 32D DNA on salivary gland chromosomes (g-j). Ch—chromocenter. Scale bar 20 μm.

The chromosome attachment region of the X-chromosome has a diffuse mesh-like structure in the middle of the left arm. It often forms unusually lateral outgrowths, which contact with the nuclear envelope. The chromosome attachment region of the 3R-chromosome in nurse cells is localized in the pericentric region, where the homologues are separate from each other. We propose to observe the morphology of these regions in other tissue with well-structured polytene chromosomes, which is convenient for analysis. DNA-probes from attachment regions of the X-chromosome and the 3R chromosome of *Anopheles messeae* were hybridized with polytene chromosomes of salivary glands cells in FISH procedure. The DNA-probes were obtained earlier by microdissection of nurse cell polytene chromosomes with following DOP-PCR [18]. The aim of the experiment is to investigate the tissue-specific morphology of nurse cell attachment regions in other tissue with polytene chromosomes. DNA-probes were located in the homologous region with a distinct chromomere arrangement and morphology. In nurse cells, the DNA-probe from the 2BC region of X-chromosome was localized, as expected, in the 2BC region, including not only the diffuse part, but additionally the bright band in 2B (Fig. 2).

**Fig 3:**
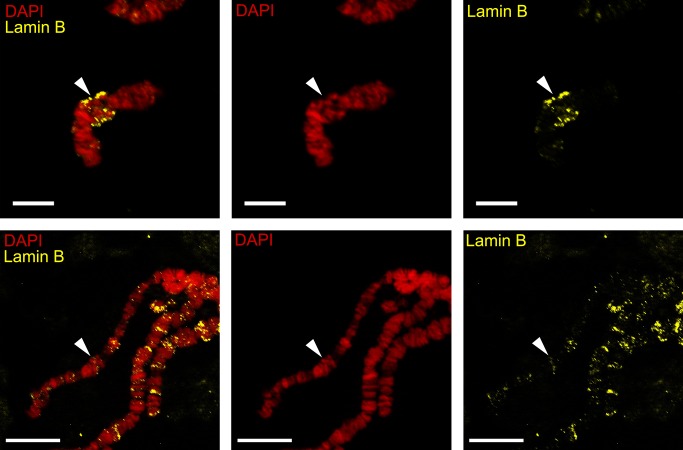
Differences in lamin localization on X-chromosomes of nurse cells (a-c) and salivary gland cells (d-f). The arrow shows the 2BC region.

In salivary gland cells this DNA-probe occupied an extended region 2A-C with seven bands and a short diffuse region in 2BC. The DNA-probe from the 32D region of the 3R chromosome was localized on the thin fibers of the nurse cell 3R chromosome pericentric region from which this probe was obtained. We found additional major signals in juxtaposed heterochromatic bands in the 32D region that are explained by the high amount of repeated sequences in the DNA-probe. In salivary glands chromosomes contact with each other via pericentric regions and form a single chromocenter. Therefore the DNA-probe of the 3R-chromosomes was localized in the chromocenter in a very compact heterochromatic block. We conclude that the morphology of chromosome regions, which conduct attachment functions in nurse cells, is distinct from the homologous region in salivary gland cells. Generally in salivary glands these regions are more compact and form more bands than in nurse cells, where it has a mesh-like structure or diffuse fibrils. These differences can be explained by the lack of chromosome attachments in 2A-C and 32D with the nuclear envelope in comparison with chromosomes in nurse cells. In order to prove this assumption we conducted immunolabeling of squashed chromosomes from nurse cells and salivary gland cells with an antibody to drosophila lamin Dm0 (Fig. 3). Lamin Dm0 is quite specific for nuclear lamina and can mark nuclear envelope debris on a squashed chromosome preparation and therefore is useful for detecting chromosome-envelope connections. This approach is not effective for detecting lamin on very thin chromatin fibrils in pericentric regions, such as 32D in nurse cells, because it tears and remains with nuclear envelope debris in the course of the chromosome preparation procedure, but it is very convenient for other, especially intercalary regions, such as 2BC [19]. We showed that X-chromosomes from both nurse cells and salivary gland cells had signals in the diffuse part of 2BC regions. The differences between these results were in the intensity (quantity) of nuclear lamina debris in these tissues. In nurse cells we found a bright signal of nuclear lamina often in the lateral position of the chromosome attachment region, but in salivary glands it is more indistinct. We assume that both in nurse cells and in salivary gland cells 2BC attaches to the nuclear envelope but with a different strength.

### Spatial localization of chromosome attachment points

The differences between chromosome organization in nurse cells with separate chromosome localization and in salivary gland cells with chromosome Rabl-orientation stimulated us to search for the same differences in other tissues of *Anopheles* mosquito.

We used DNA-probes from investigated chromosome regions always localized on the nuclei periphery. The absence of well-structured polytene chromosomes in many cell types complicates this task. Therefore we proposed to investigate the distance between two points on the nuclear periphery localized on different chromosomes and having evident contacts with the nuclear envelope in nurse cells. In order to accomplish this task we used DNA-probes from the chromosome attachment region of the X-chromosome and 3R chromosome as per the previous experiment. DNA-probes were hybridized with intact nuclei of several tissues. For the experiment, cells were selected with differing ploidy levels (diploid and polyploid cells) type polyteny (classic polyteny and latent polyteny), lineage (germline cells and somatic cells), potential for further development (terminally differentiated and stem cells). Thus we used the following cell systems: 1) ovarian nurse cells (terminally differentiated germline cells with classical polytene chromosomes), 2) salivary gland cells of larvae (terminally differentiated somatic cells with classical polytene chromosomes), 3) follicular epithelium cells (terminally differentiated somatic cells with a latent polyteny) and 4) imaginal discs cells, (somatic pluripotent system cells with a diploid set of chromosomes).

We found that in the main part of investigated nuclei in all cell types the 2BC and 32D regions are localized near the nucleus periphery. The structure of these regions was specific in each tissue. In the nurse cells we found a single (very rarely, a double) signal corresponding to the 2BC chromosome region. The 32D region was presented in such cell types with double signals. It may be explained by homologous diversity, which is common for the pericentric region of the 3R chromosome (Fig. 2), and often in the 2BC region of the X-chromosome in An. messeae. In salivary glands we observed only two compact signals. In follicle epithelium cells 32D has a very extended and mesh-like morphology in comparison with 2BC, which has a more compact one. In imaginal discs both signals were local and compact with an irregular shape. Thus the chromosome regions of each cell type have a distinct morphology, probably connected with its function in chromosome attachments.

### Spatial interposition of nurse cell chromosome attachment points in different cell types of Anopheles messeae

The specific spatial organization of whole polytene chromosomes and the chromocenter in different tissues of Drosophila has been shown earlier [26]. However general principles of chromosome architecture in connection with insect ontogenesis are still unknown. In spite of the unraveling organization of the polytene chromosomes, they may be an adequate model of the interphase nucleus, as has been demonstrated in recent works [27, 28]. We obtained results on the spatial localization of chromosome attachment regions from four larvae and imago tissues. For every cell the angle between imaginary vectors was measured, i.e. that line from the center of the nucleus to each attachment point of the X-chromosome and 3R chromosome (Tables A-E in S1 File). This approach, we believed, was more convenient for such an investigation due to the fact that the angle value does not depend on the nucleus shape significantly, but allows a comparison of tissues with different cell nuclei sizes.

The samples underwent a statistical comparison, which showed that only salivary gland cells and follicle epithelium cells have no significant difference in chromosome interposition in *An*. *messeae* (Table 1).

**Table 1:** Comparison of X-chromosome and 3R chromosome regions interposition in different tissues by *t*-test (*p*-values).

	*An*. *atroparvus* NC	*An*. *messeae* NC	*An*. *messeae* IDC	*An*. *messeae* SGC
An. atroparvus NC	-	-	-	-
An. messeae NC	0.334537	-	-	-
An. messeae IDC	-	**0.000101**	-	-
An. messeae SGC	-	**0.000000**	**0.044429**	-
An. messeae FEC	-	**0.000000**	**0.000336**	0.159166

NC- nurse cells

IDC—imaginal discs cells

SGC—salivary glands cells

FEC- follicle epithelium cells

*p*-Values are bold, if differences are significant (*p*<0.05)

**Fig 4:**
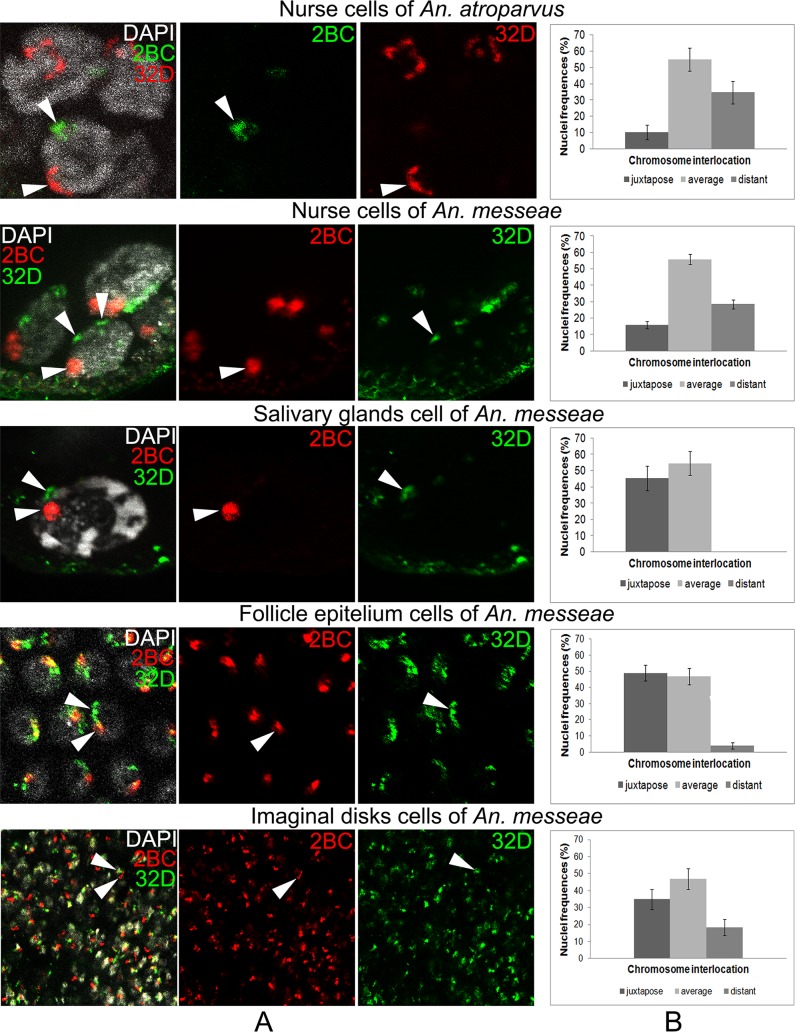
Interposition of the X-chromosome region (2BC) and 3R chromosome region (32B) in the nucleus space (A) and the frequencies of nuclei with juxtaposed, average and distant interposition of these regions (B).

The frequencies of nuclei with different angle values were calculated for each cell type. In the salivary glands cells and follicle epithelium cells the chromosomes were located significantly closer to each other in comparison with imaginal discs cells and especially with nurse cells (Fig. 4). These data can be explained by the connection of the X-chromosome and 3R chromosome in the chromocenter, which restrict their spatial separation in the nucleus. Chromosome 2, chromosome 3 and X-chromosome connect with each other in the centromeric region and confirm this proposal (Fig. 2). A remarkable single heterochromatic block in follicle epithelium nuclei may also be formed by the chromocenter (Fig. 4).

For the nurse cells we found another situation, when nuclei with a large distance between chromosomes were found with a higher frequency of about 30%. Therefore terminally differentiated somatic cells, such as salivary gland cells and follicle epithelium cells have a specific spatial organization and distinct terminally differentiated germ line cells—nurse cells. It is interesting that the imaginal discs cells have an intermediate chromosome arrangement. Imaginal discs cell nuclei show high frequency of juxtaposed and average interposition of chromosomes as was shown for other somatic cells, as well as a relatively high percentage of chromosomes with a distant interposition, similar to nurse cells (Fig. 4). These results demonstrate the important role of chromosome interposition in organism development.

### Comparison of spatial interposition of chromosome attachment points in the nurse cells of *Anopheles atroparvus* and *Anopheles messeae*

Remarkable interspecific differences in spatial organization of chromosomes in malaria mosquitoes nurse cells stimulated us to compare the interposition of chromosome attachment points in two sibling species—*Anopheles atroparvus* and *Anopheles messeae*. We assumed that in different species with different characteristics of chromosomes attachments we would find a different interposition of chromosome attachment points. For this experiment we additionally obtained DNA-probes from the pericentric regions of the X-chromosome and 3R chromosome of *Anopheles atroparvus* [29]. Followed by labeling these DNA-probes were hybridized with intact nurse cells of *Anopheles atroparvus*. The result of this hybridization was processed as described above. The distribution of frequencies between angle values for nurse cells of *Anopheles atroparvus* and *Anopheles messeae* has no statistical differences. We found a very high frequency of nuclei with an average distance between attachment points, but also a remarkable percentage of nuclei with distant and juxtaposed interposition. It is very important to note that this feature is common for ovarian nurse cells of both species, which were analyzed.

## Discussion

**Fig 5:**
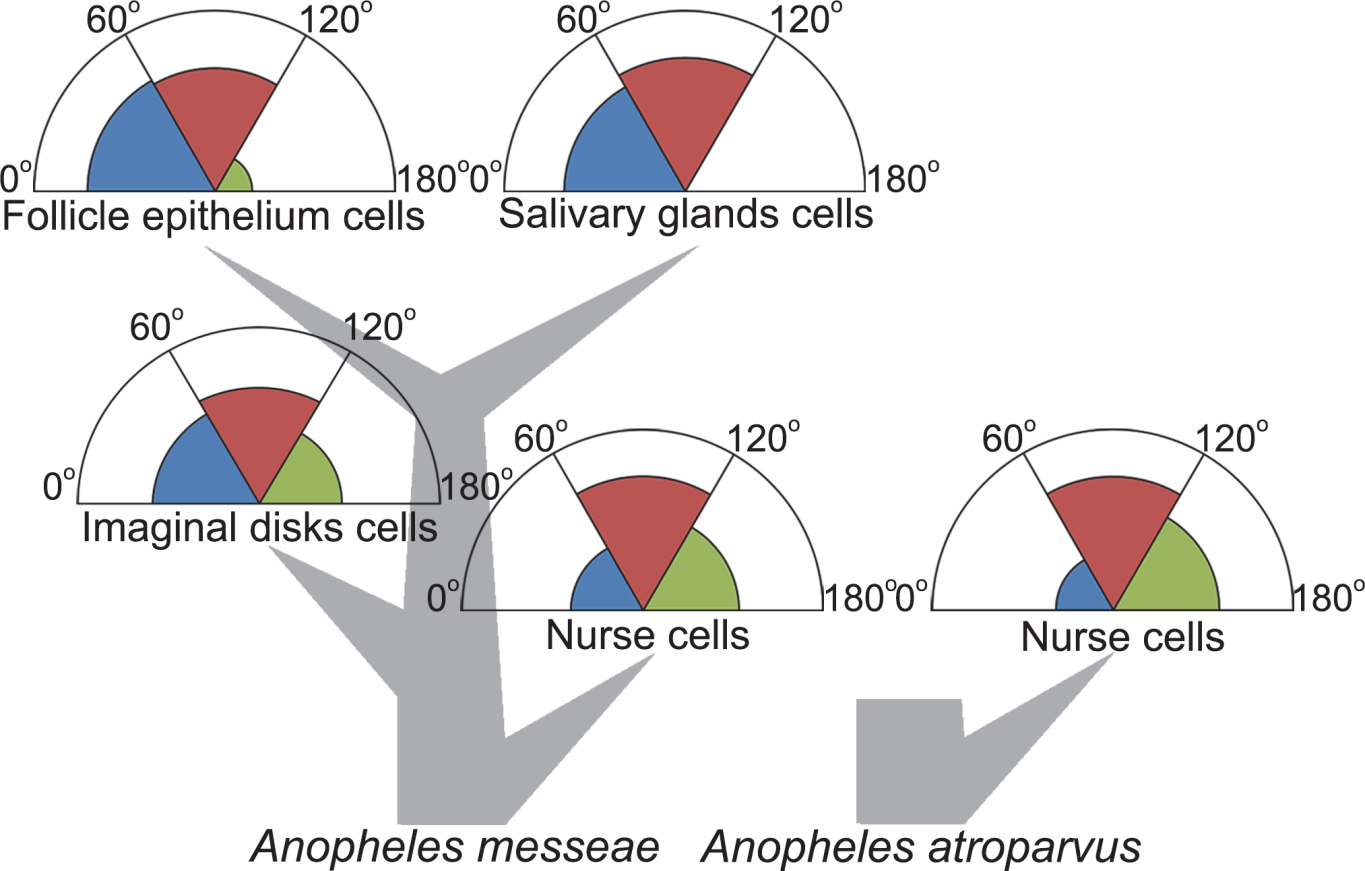
Summaries for the X-chromosome and 3R-chromsome tissue-specifics in connection with developmental trees for *An*. *messeae* and *An*. *atroparvus*.

Our study found tissue-specific differences in the morphology and interposition of the X-chromosome and 3R chromosome regions, which provide attachments to the nuclear envelope in nurse cells. Classical polytene chromosomes, developed both in nurse cells and in salivary gland cells of malaria mosquitoes, are very useful for researching tissue-specific features of the genome organization. We showed remarkable differences between the chromomere organization of X-chromosome attachment regions and especially of 3R chromosomes. In spite of the contacts with nuclear lamina, these regions revealed a more compact structure in salivary gland cells, but in nurse cells it was diffuse. It is probable, that the reason for these differences is in tissue-specific strategies of attachments. Chromosome attachment regions have minor contacts in salivary gland cells, but in nurse cells they tend to occupy a greater area on the inner nuclear envelope, probably in order to have a stronger connection. These tissue-specific differences become brighter when coupled with a global chromocenter or non-chromocenter organization of the nucleus. The study of such features as chromosome interposition seems more difficult in cell types without polytene chromosomes. However we used 3D-FISH for analyzing the chromosome arrangement inside the nuclei of several tissues, comparing the interposition of the 2BC region from the X-chromosome and 32D from the 3R chromosome. These regions obviously involve attachment chromosomes to the nuclear envelope in nurse cells and probably in salivary gland cells, but we do not know about any influence on chromosome-nuclear envelope relations in other cell types. In spite of that, the interposition of two regions from two distinct chromosomes could show the general chromosome arrangement and its similarity to chromocenter or non-chromocenter models, which we observed in salivary gland cells and nurse cells respectively. We found that the 2BC region and 32D region were localized near the nuclear periphery in all cell types, which may explain its heterochromatic nature or/and indirect support its involvement in connection with the nuclear envelope. All the differences in the interposition of chromosome regions were not absolute, but statistical. A simple division of samples into three groups showed statistical differences in chromosome interposition characteristics (Fig. 4B). It is very important that only one comparison of the follicle epithelium and salivary gland cells, belonging to terminally differentiated cells of the somatic system, had no statistical differences in *An*. *messeae*. The type of polyteny does not affect chromosome interposition, because salivary gland cells and follicular epithelium cells have classic and latent types of polyteny respectively. This proposal is proved by the remarkable differences in chromosome interposition in nurse cells and salivary glands cells with similar classical polyteny. Therefore we found three distinct groups in the chromosome interposition arrangement—terminally differentiated somatic cells, imaginal discs cells and nurse cells (Fig. 5). Follicle epithelium cells and salivary gland cells had either no or very low frequencies of distant X-chromosome and 3R chromosome interposition whereas in nurse cells such nuclei were observed at a rate of about 30%. Imaginal discs cells have an intermediate position in the system of differentiation and, according to this system, chromosome spatial interposition has characteristics from chromocenter-like and non-chromocenter-like systems. In the somatic system chromosome architecture tends to have a chromocenter-like model and differs strongly from that of a nurse cell. Germ-line cells, like nurse cells, may develop in another way and form a specific chromosome architecture. It can be explained by the exclusive germ-line cells mission—to transfer epigenetical information to the next generation, or specific conditions for gene expression of nurse cells that are required for ovary feeding. These suggestions may be very speculative and require detailed analysis, but our result demonstrates that chromosome arrangement can play an important role in differentiation processes.

An analysis of the spatial interposition of chromosome attachment points in nurse cells in another mosquito, *An*. *atroparvus*, showed similar results to those obtained from the nurse cells of *An*. *messeae* (Fig. 4B). It suggests that nurse cell chromosome interpositions are not species-specific in contrast to chromosome and nuclear envelope interconnections. Therefore we can conclude that chromosome interposition is not as important for species identity as for nurse cell function.

Our study aimed to search for inter-tissue and inter-specific differences in the X-chromosome and 3R chromosome interposition in the nurse cells of malaria mosquitoes. As a result, we found tissue-specific differences that are probably connected with gene expression and which influence the differentiation process. A distinct chromosome arrangement and chromosome attachment region system in nurse cells provides evidence of the unusual nuclear architecture of germ-line cells. However we do not know about chromosome organization in other cells from the generative system of malaria mosquitoes. We plan to detect the development stage, when strategies of chromosome arrangement in the nucleus space evolve in two differing ways that form somatic and generative cell systems.

## Supporting Information

S1 File File includes Tables A–E. Table A: Calculation of the average angle between imaginary vectors, that line from the center of nucleus to X-chromosome signal and to 3R chromosome signal in *Anopheles atroparvus* nurse cells. Table B: Calculation of the average angle between imaginary vectors, which line from the center of nucleus to X-chromosome signal and to 3R chromosome signal in *Anopheles messeae* nurse cells. Table C: Calculation of the angle between imaginary vectors, which line from the center of nucleus to X-chromosome signal and to 3R chromosome signal in *Anopheles messeae* salivary glands cells. Table D: Calculation of the angle between imaginary vectors, which line from the center of nucleus to X-chromosome signal and to 3R chromosome signal in *Anopheles messeae* follicle epithelium cells. Table E: Calculation of the angle between imaginary vectors, which line from the center of nucleus to X-chromosome signal and to 3R chromosome signal in *Anopheles messeae* imaginal discs cells.
(XLSX)
